# Predictive value of grade point average (GPA), Medical College Admission Test (MCAT), internal examinations (Block) and National Board of Medical Examiners (NBME) scores on Medical Council of Canada qualifying examination part I (MCCQE-1) scores

**Published:** 2016-03-31

**Authors:** Banibrata Roy, Ira Ripstein, Kyle Perry, Barry Cohen

**Affiliations:** 1 Department of Community Health Sciences, College of Medicine, University of Manitoba; 2 Division of Undergraduate Medical Education, College of Medicine, University of Manitoba; 3 Department of Pathology, College of Medicine, University of Manitoba

## Abstract

**Background:**

To determine whether the pre-medical Grade Point Average (GPA), Medical College Admission Test (MCAT), Internal examinations (Block) and National Board of Medical Examiners (NBME) scores are correlated with and predict the Medical Council of Canada Qualifying Examination Part I (MCCQE-1) scores.

**Methods:**

Data from 392 admitted students in the graduating classes of 2010–2013 at University of Manitoba (UofM), College of Medicine was considered. Pearson’s correlation to assess the strength of the relationship, multiple linear regression to estimate MCCQE-1 score and stepwise linear regression to investigate the amount of variance were employed.

**Results:**

Complete data from 367 (94%) students were studied. The MCCQE-1 had a moderate-to-large positive correlation with NBME scores and Block scores but a low correlation with GPA and MCAT scores. The multiple linear regression model gives a good estimate of the MCCQE-1 (R2 =0.604). Stepwise regression analysis demonstrated that 59.2% of the variation in the MCCQE-1 was accounted for by the NBME, but only 1.9% by the Block exams, and negligible variation came from the GPA and the MCAT.

**Conclusions:**

Amongst all the examinations used at UofM, the NBME is most closely correlated with MCCQE-1.

## Introduction

Experts have long debated about the appropriateness of various formats of assessment of undergraduate medical students. Starting from their admission to a Canadian medical school based on the MCAT and GPA scores, students undergo several pre-clerkship (Year 1 and Year 2 Block examinations) and clerkship examinations (Year 3 and Year 4), followed by the final licentiate examination MCCQE-1. Although NBME subject examinations at the end of clerkship rotations are administered in most of the Canadian medical schools, many schools do not use them. The primary objective of this article is to address the important issue of how the MCCQE-1 can be explained by various examination scores, especially NBME scores at the University of Manitoba (UofM). This study will explore the relationship between MCCQE-1 and the explanatory variables such as NBME scores, Year 1 and Year 2 Block scores, GPA and MCAT scores, thereby evaluating the comparative importance of these local assessment programs with an external standardized exam, MCCQE-1. The knowledge of this relationship will help to identify at-risk students in that specific assessment which is most closely related to MCCQE-1, thereby providing support to the students at risk of failing the MCCQE-1. This study makes a strong case to introduce NBME examinations in all the medical schools across Canada.

Similar to most medical schools in Canada and the United States, the UofM offers a 4-year medical curriculum which is divided into a pre-clerkship phase (first two years) and a clerkship phase (last two years). Each academic year of pre-clerkship is composed of three Blocks, at the end of which, an examination consisting of multiple choice and short answer questions is administered. The Year 1 Block average is the mean performance of Blocks 1, 2 and 3 while the Year 2 Block average is the mean performance of Blocks 4, 5 and 6. The Combined Year Block average is the mean performance of Year 1 and Year 2 Block averages.

The 8 core clinical rotations of 48 weeks, comprising of rotations in Internal Medicine, Surgery, Paediatrics, Psychiatry, Obstetrics/Gynaecology, Family Medicine, Emergency Medicine, and Anaesthesia take place in the third year of medical school at UofM. The fourth year comprises of 14 weeks of Electives followed by 3 weeks of CaRMS and then 11 weeks of Transition to Residency (TTR).

National Board of Medical Examiners (NBME) subject examinations are administered at the end of the clerkship rotations in each of Internal Medicine, Surgery, Pediatrics, Psychiatry and Obstetrics/Gynecology. Combined NBME is the mean performance of these 5 major NBME examinations.

The MCCQE-1 is a summative examination that all medical graduates must pass as a component to obtaining licensure in Canada. Administered by the Medical Council of Canada, MCCQE-1 is a computer-based test comprising of two sections - Multiple Choice Questions (MCQ) (worth 75%) and short-menu and short-answer case questions - Clinical Decision Making (CDM) (worth 25%).^1^ The standard score scale is from 50–950 with a minimum passing score of 390.

The Medical College Admission Test (MCAT) examines the applicant’s knowledge and problem solving skills in three multiple choice sections: Physical Sciences (Physics, Chemistry); Biological Sciences; and Verbal reasoning. This is a pre-admission test taken by all medical school applicants at the UofM.

Grade point average (GPA) is the adjusted score for all undergraduate courses completed.

This study was conducted using the performance data of the medical students from the UofM graduating classes of 2010, 2011, 2012 and 2013. The purpose of this study was to determine whether the two yearly block average scores and the five NBME subject scores had any significant correlation with the MCCQE-I scores and to determine whether the pre-admission test measures GPA and MCAT had any significant correlation with the post admission performance of Block, NBME and MCCQE-1.

NBME subject examinations are used by many Canadian medical schools to evaluate students during their clerkship rotations. Although several studies have shown a strong correlation between NBME subject scores and United States Medical Licensing Examination (USMLE) Step 1 and Step 2 scores,^2^–^7^ there is little published research comparing NBME with MCCQE-1.^8^

According to the theory of “Context Specificity,” academic performance in one subject/context does not necessarily correlate strongly with the performance in another subject/context for a given student. Using this framework, the following four hypotheses were stated:

H_1_: Performance of individual NBME subject exam scores will have a small-to-moderate correlation with overall performance on MCCQE-I examinations.
H_2_: The combination of all NBME subject exam scores will account for a moderate-to-large amount of variance in MCCQE-I scores since the questions and syllabus topics of those NBME subjects match to a large extent with MCCQE-I.
H_3_: The two yearly (Year 1 and Year 2) Block average scores will have a small correlation with the MCCQE-I scores since the basic science courses in the first two years are quite different from the MCCQE-I syllabus content.
H_4_: MCAT and GPA score performances, based on courses taken prior to medical admission will not correlate to a great degree with MCCQE-I scores, as this examines clerkship material quite different from the pre-medical courses.

## Methods

### Setting and sample size

The initial study sample consisted of data collected from 392 students who were admitted to graduating classes of 2010, 2011, 2012 and 2013. The final analysis was done using the complete data from only 367 students, which represented 94% of the initial population. Giving a year wise break-up, these 367 students represent 89, 85, 95 and 98 students respectively for the years 2010, 2011, 2012 and 2013. This overall decrease of 25 students was due to various reasons. Some students dropped out of the program or took a leave of absence after taking some of their examinations, while some have failed or skipped examination(s) due to illness or family emergency, thus resulting in incomplete data. Moreover, all of the recorded data are first time scores in NBME and MCCQE-1 and no remediated scores were included in the process. Since the missing cases account for just 6% of the initial population and seeing the trend of the recorded data in those missing cases, it is assumed that they would not impact the results of this study to any considerable extent.

### Study design variables and statistical analysis

Data relating to the students’ MCAT, GPA plus the performance on their six Block and five NBME examinations were collected for the four graduating classes of 2010–2013. Finally MCCQE-1 scores were collected for all the students.

The primary outcome variable (dependent variable) was the MCCQE-1 score while the explanatory variables (independent variables) were the five NBME subject exam scores, Year 1 block average, Year 2 block average, MCAT and GPA. Year-wise demographic characteristics were studied to assess the gender ratio and mean MCAT and GPA scores of admitted students. Descriptive statistics depicting the minimum, maximum, mean and standard deviation (SD) scores of all the students for the study variables were determined, followed by Karl Pearson’s bivariate correlation coefficient matrix between MCCQE-1 score and several explanatory variables.

The bivariate correlation matrix quantifies the degree to which two variables are related. Correlation does not fit a line through the data points. The computation of the pair-wise correlation coefficient (*r*) tells us how much one variable tends to change when the other one does. When *r* is positive, there is a trend that performance in one variable goes up as the performance in the other one goes up. Linear regression finds the best line that predicts the dependent variable from a set of independent variables. In other words, correlation helps to determine whether students who are good at one subject tend to be good at another subject as well, while regression allows one to determine whether the marks in one subject can be predicted for a given mark in another subject. Taking advantage of the correlation matrix which illustrated a very negligible pairwise correlation of MCAT and GPA with MCCQE-1 (reason for not considering them in the multiple linear regression), a multiple linear regression model was fitted to the data to estimate the probable MCCQE-1 score based on the NBME combined and the combined yearly block average score. *R*^2^ was computed to find the overall fit of the model. Finally, stepwise linear regression was conducted to further investigate the association between the explanatory variables and the only outcome variable, MCCQE-1.

Prior to data analysis, an examination of the regression model assumptions indicated a satisfactory level of homoscedasticity, linearity and normality. Multicollinearity is a problem that occurs with regression analysis when there is a high correlation of at least one independent variable with a combination of the other variables. Some people suggest to look at the correlation matrix and see if any independent variable correlate above some level (may be 0.75 or even 0.90) with one another. Ideally, this view doesn’t go far enough to recommend a presence of multicollinearity since intuitively this correlation describes a bivariate relationship, whereas collinearity is a multivariate phenomenon. Certainly if one has variables correlated above 0.90, one should not include both in the regression equation. But even with values around 0.70, one should proceed. Although high pair-wise correlations could be the first indicator of collinearity problems, one should not confirm the presence of multicollinearity just by examining the correlation matrix. One should compute the collinearity diagnostics comprising of the Tolerance (T) statistic and the Variance Inflation Factor (VIF). To compute a Tolerance statistic for an independent variable, a multiple regression is performed with that variable as the new dependent variable and all of the other independent variables in the model as independent variables. Since *R*^2^ is the amount of variance in a dependent variable in a multiple regression explained by a combination of all independent variables, the tolerance statistic T is equal to 1-*R*^2^. T < 0.20 is generally considered cause of concern. It means that the multiple correlation of the other independent variables with the independent variable is at least 0.90 (because 0.9 × 0.9 = 0.81).

The other statistic, Variance Inflation Factor (VIF) is the reciprocal of the tolerance statistic. A VIF of value greater than 5 is generally considered evidence of multicollinearity. In this study, T > 0.2 and VIF < 5 indicated absence of multicollinearity. Moreover, if the sole purpose of the regression analysis is prediction or forecasting (as in this study), then multicollinearity is not a serious problem while in cases of reliable estimation of parameters (which this study does not aim to do), higher pairwise correlation coefficients can pose serious issues.

Another important regression model assumption is the test for “Residual Analysis.” Because a linear model is not always appropriate for the data, one should assess the appropriateness of the model by defining residuals and examining residual plots. The residual (e) is the difference between the observed value (y) and the predicted value (ŷ), i.e. e = y – ŷ, with e = 0. A residual plot is a graph of the residual on the vertical axis and each independent variable (x) on the horizontal axis. If the points in a residual plot are randomly dispersed around the horizontal axis, a linear regression model is appropriate for the data; otherwise, a non-linear model is more appropriate. Alternatively, a test for linear first order autocorrelation using the Durbin-Watson test can be performed to check whether the residual terms are correlated with each other. The Durbin- Watson test statistic ranges from 0 to 4. A value close to 0 indicates strong positive correlation while a value of 4 indicates strong negative correlation. As a general rule, the residuals are uncorrelated (no autocorrelation) if the Durbin-Watson statistic is approximately 2 (an acceptable range is 1.50 – 2.50). For this study, the Durbin-Watson test statistic is 1.91, approximately close to 2, indicating no serial correlation.

Based on the finding that NBME results were more correlated to MCCQE-1 than the yearly Block average scores, individual NBME subject exam scores were first entered in the stepwise regression model followed by yearly Block average scores. Next, the pre-admission scores MCAT, GPA were entered. Significance was set at *p* < 0.05. The entire statistical analysis was done using SPSS version 21.0 (IBM SPSS Statistics).

### Reliability and validity of measurement instruments

An assessment of data integrity plays a significant role in determining if the data are appropriate for statistical analysis. The reliability of a measure, explained as the basis of consistency and accuracy, can be described as the extent to which the instrument yields the same results on repeated trials. The average GPA scores of the UofM students in the graduating classes (2010–2014) of study was consistently measured around 4 while the average MCAT scores of these students was measured around 10.50. For the measuring instrument of Block average, Year 1 Block average had a consistent measure of around 76.81 while Year 2 Block average has a consistent measure of around 75.34 over the four years of study. Although the admission criteria (a minimum score for GPA and MCAT) was consistent across the graduating classes, this analysis of the mean GPA and MCAT scores provides a measure of the fairly consistent intellectual capabilities of the admitted students in the four graduating classes, which gets further reinforced by the students’ consistent averages in the Block scores. The reliability of the outcome measure “MCCQE-1” is best captured by studying the internal consistency using Cronbach’s alpha, which measures the degree to which the independent variables GPA, MCAT, Block and NBME affect the dependent variable MCCQE-1. When assessing the internal consistency reliability, one is not actually assessing the reliability of the measurement tool, but the reliability of the study data in the context of the measurement tool. There is a general agreement that the Cronbach’s alpha rating must be at least 0.70 to be considered acceptable,^9^–^11^ although there are many reports that recommend a higher number with acceptable alpha values ranging from 0.70 to 0.95.^12^ In this study, the Cronbach’s alpha rating is an acceptable internal consistency of 0.82.

An examination of a study measurement tool’s validity considers whether a tool collected sufficient data to infer that the trait or construct being surveyed was the actual construct measured. GPA, MCAT and Block scores appear to have face validity since they are inherently designed to measure the traits, capabilities, and knowledge that the candidates require to complete their program of study. For the purpose of this study however, these scores lacks content validity as they are heavily based on basic science and pre-clinical content, and are therefore different from NBME subject examinations and the MCCQE-1 examination which measure candidates’ knowledge in the specialised clerkship rotations. Therefore, by undertaking these measurements of reliability and validity, the required checks of data integrity have been satisfied.

In the current study, the Sensitivity (= a / (a + c) × 100%) is the ability of the model to correctly identify students who pass the MCCQE-1 exam. The Specificity (= d / (b + d) × 100%) is the ability of the model to correctly identify students who fail the MCCQE-1 exam. The Positive Predictive Value, PPV (= a / (a + b) × 100%) is the percentage of students predicted to pass the MCCQE-1 exam based on the regression model who have actually passed the MCCQE-1 exam. The Negative Predictive Value, NPV (= d / (c + d) × 100%) is the percentage of students predicted to fail the MCCQE-1 exam based on the regression model who have actually failed the MCCQE-1 exam. The Accuracy (= (a + d) / (a + b + c + d) × 100%) of the model discusses how often the model is correctly predicting the Pass/Fail in MCCQE-1 exam.

## Results

After removing the 25 students who missed on some of the requisite exams on schedule, this study was based on 367 (94%) students out of an initial sample of 392 students in the graduating classes of 2010–2013.

Table 1 gives the demographic data for all 367 students. The male:female ratio was almost evenly balanced for all four graduating classes. The pre-medical performance measures, GPA and MCAT have an overall mean score of 4.09 and 10.52, respectively. The range of the mean values of GPA for all four graduating classes was 4.04 to 4.17; while that of MCAT was 10.32 to 10.68.

Table 2 provides descriptive statistics for all 367 students showing mean, SD and range (minimum and maximum) for the individual NBME subject exams, Combined NBME, Year 1 Block Average, Year 2 Block Average, Combined Year Block Average, MCCQE-1, MCAT and GPA. The Combined NBME had a mean value of 76.51 (SD=6.96) while the Combined Year Block average had a mean value of 76.08 (SD = 5.66).

Table 3 presents Karl Pearson’s bivariate correlation coefficient of the explanatory variables and MCCQE-1 scores, with significance reached at *p* < 0.001. Correlation analysis between the Combined NBME and MCCQE-1 was found to be quite strong at 0.766 while that between Combined Year Block Averages and MCCQE-1 was found to be 0.683.

As shown, MCCQE-1 scores had moderate-to-high positive correlations with all individual NBME subject exam scores (*r* = 0.617 – 0.676) and also Year 1(*r* = 0.617) and Year 2 (*r* = 0.664) Block Averages; while the correlation of MCCQE-1 with the pre-admission test measures MCAT (*r* = 0.319) and GPA (*r* = 0.372) were weak. In addition, NBME subject exam scores for the different clerkships were positively correlated with each other (*r* = 0.602 – 0.686) and with the Year 1 (*r* = 0.605) and Year 2 (*r* = 0.676) Block averages.

Table 4 (next page) gives the SPSS output of the Multiple Linear Regression model to estimate the likely MCCQE-I score for given combined NBME score and Combined yearly Block average score. The Analysis of Variance (ANOVA) table which shows the significance of the *F* value at least at the alpha level less than 0.001 indicates that the model is statistically significant.

The Model Summary gives the coefficient of determination, *R*^2^ with a value of 0.604 which means that 60.4% of the variation in MCCQE-1 has been explained by the two independent variables. Moreover, the *t*-test with significant values less than 0.001 in the Coefficients table indicates the importance of the two independent variables in the model.

Using the unstandardized B coefficients, we write the Multiple Linear Regression model as:

MCCQE_1=-128.11+6.32 (NBME_Combined)+2.66 (Combined_Year_Block_Average)

Table 5 (next page) shows the stepwise linear regression model in three steps to determine which explanatory factors contributed more towards the total amount of variation in MCCQE-1. The significant values of the *F* test in each of the three steps at an alpha level less than 0.001, as shown in the ANOVA table indicates that the results are significant.

In the first step of the regression model, as shown in the coefficients table, NBME subject exam scores of Internal Medicine, Surgery, Pediatrics, Psychiatry, and Obstetrics and Gynecology explained 59.2% of the variance in MCCQE-1 scores. The scores of Year 1 and Year 2 Block averages, when entered together as a Block in the second step of the regression model, accounted for an additional 1.9% of variance. Lastly, when the MCAT and GPA were entered as a Block in the third step of the regression model, it accounted for only 0.2% which shows that they have practically no unique contribution in predicting MCCQE-1 performance. The *R*^2^ of the final model was 0.613 indicating that 61.3% of the total variation in MCCQE-1 can be explained by all the explanatory variables.

Table 6 shows the evaluation of the prediction effects of the multiple regression model to study sensitivity, specificity, positive predictive value, negative predictive value and the accuracy of the model.

## Discussion

Considering that passing the MCCQE-1 is a necessary component for physicians to obtain medical licensure and that many of the medical schools across Canada utilize the NBME subject examinations, there is value in demonstrating a relationship between the MCCQE-1 examinations and the NBME subject examinations. The sample data collected from 367 students in the graduating classes of 2010–2013 at the University of Manitoba suggests:

1. A moderate-to-high degree of correlation within the NBME subject exam scores.
2. A moderate-to-high degree of correlation between the NBME subject exam scores and MCCQE-1 exam scores.
3. A low degree of correlation between MCCQE-1 and MCAT/GPA.

Also, there is a relatively high correlation between MCCQE-1 and NBME combined score as compared to Block average scores.

The stepwise regression analysis was quite interesting. NBME subject exam scores explained about 60% of the variation in MCCQE-1 exams, whereas Block exam scores was only 2% and MCAT/GPA was 0.2%. The most likely reason for this is that NBME and MCCQE-1 exams assess similar content, namely a candidate’s knowledge in the specialized clerkship rotations, while Block examinations and MCAT/GPA essentially asses pre-clinical learning.

One of the strengths of this study is the large sample of students with access to each score throughout their medical school program, as well as their MCAT score and their undergraduate GPA. Moreover, the data integrity has been checked with reference to the various regression model assumptions, especially that of multicollinearity and residual analysis.

This study has several limitations. The data in this study has been collected from a single academic institution (University of Manitoba), but it would have been a stronger study if the data could have been included from several other institutions. Nonetheless, similar studies can be done in other institutions also. Since NBME and MCCQE-1 are national standardized exams, these correlations will likely be reproducible in other institutions. The MCAT and GPA results do not show any significant contribution to MCCQE-1 and hence a multiple regression equation to predict MCCQE-1 has not been involved. Although 60% of the variance in MCCQE-1 performance is being explained by NBME subject scores, it is important not to over-interpret their contributions, as nearly 40% of variance was still unaccounted for. Other factors that could affect the performance in the MCCQE-1 exam, which were not considered in this study include test-taking strategies, fatigue, anxiety, clerkship length and /or timing in the academic year^13^–^18^ as well as the content of electives. Furthermore, it is important to note that the specific variables and the order in which these were entered into the Stepwise regression model can sometimes affect the percentage of variance explained by those variables to MCCQE-1. However, in order to specifically determine the variance accounted for by a combination of Block and NBME exams, the NBME is elected to enter the variables first due to their strong correlation with MCCQE-1 followed by the Yearly Block averages.

In conclusion, the findings of the study strongly suggest that NBME subject exams exhibited moderate to large positive correlation with MCCQE-1 scores and also explain a considerable amount of variance in the performance of MCCQE-1 exams at the University of Manitoba. Since NBME is most closely related to MCCQE-1, undergraduate medical educators interested in predicting performances on national standardized examinations could use this information to plan remediation for students who have struggled on their NBME examinations. This study could also assist individual students to take pro-active remediation measures, by seeking extra learning opportunities and assistance for subjects in which they had poor NBME exam results. Post-graduate medical educators or program directors may be interested in using this NBME information to aid in their selection of residents. Finally, this research initiative may serve to encourage more medical schools in Canada to undertake these standardized NBME examinations in pursuing the broader goal of improving medical education in Canada. This broad objective can be achieved specifically by preparing students with extra help prior to challenging the MCCQE-1 based on their NBME scores wherein schools will want to add them as measures to predict the success on the MCCQE-1.

Since the Medical Council of Canada (MCC) assesses the competence of physicians seeking to practise medicine in Canada by verifying their core skills and knowledge, patients can be confident that their physicians meet the same demanding, consistent standards across the country and hopefully higher MCCQE-1 scores translates into better enhanced knowledge for the future physicians in providing better patient care.

## Figures and Tables

**Table 1:** Student characteristics (n=367)

Year	Gender	Frequency	%	Total (367)	Mean GPA score	Mean MCAT score
2010	F	42	47.2	89	4.04	10.32
	M	47	52.8			
	Total	89	100.0			
2011	F	42	49.4	85	4.08	10.49
	M	43	50.6			
	Total	85	100.0			
2012	F	40	42.1	95	4.06	10.58
	M	55	57.9			
	Total	95	100.0			
2013	F	50	51.0	98	4.17	10.68
	M	48	49.0			
	Total	98	100.0			

**Table 2:** Descriptive statistics for the NBME subject exam scores, yearly block averages and the MCCQE-1 scores (n=367)

Variable	Minimum	Maximum	Mean	SD
Medicine	54	99	75.82	7.95
Surgery	45	99	74.75	8.93
Psychiatry	58	99	79.73	7.86
Obs./Gyn.	50	99	76.1	8.41
Pediatrics	52	99	76.13	8.31
NBME combined	59.4	99	76.51	6.96
Year 1 block average	60.47	90.85	76.81	5.22
Year 2 Block Average	54.7	93.12	75.34	7.12
Combined year block average	58.94	90.57	76.08	5.66
MCCQE-1	353	850	543.59	71.18
GPA	3.28	4.5	4.09	0.25
MCAT	7.25	13.2	10.52	0.99

**Table 3:** Karl Pearson’s Bivariate Correlation Coefficient with significance at *p* < 0.001

	Medicine	Surgery	Psychiatry	Obst_Gyn	Pediatrics	NBME_Com	Year_1_Bl_Ave	Year_2_Bl_Ave	Com_Year_Bl_Avg	MCCQE_I	MCAT	GPA
Medicine	1	0.629	0.602	0.649	0.686	0.846	0.640	0.598	0.671	0.636	0.340	0.353
Surgery		1	0.604	0.564	0.682	0.835	0.619	0.603	0.664	0.623	0.316	0.380
Psychiatry			1	0.585	0.644	0.813	0.576	0.551	0.612	0.617	0.239	0.351
Obst_Gyn				1	0.663	0.827	0.625	0.568	0.645	0.664	0.260	0.387
Pediatrics					1	0.876	0.659	0.631	0.701	0.676	0.272	0.382
NBME_Com						1	0.744	0.703	0.785	0.766	0.340	0.442
Year_1_Bl_Ave							1	0.677	0.886	0.656	0.363	0.483
Year_2_Bl_Ave								1	0.941	0.605	0.287	0.398
Com_Year_Bl_Ave									1	0.683	0.347	0.473
MCCQE_I										1	0.319	0.372
MCAT											1	0.417
GPA												1

**Table 4:** Multiple linear regression

One way ANOVA of MCCQE-1 by combining Year Block average and NBME combined									
Source				Sum of Sq.	d.f.	Mean Sq.		*F*-Value	*P*-value
Between groups				1119876.08	2	559938.04		277.56	< 0.001
Within groups				734332.62	364	2017.40			
Total				1854208.69	366				

**Model Summary**									

**Model**	***R***	***R*^2^**		**Adjusted** ***R*^2^**		**Std. Error**		**Durbin-Watson**	
1	.78	0.60		0.60		44.92		1.910	

**Coefficients**									
Model			Unstandardized Coef.		Std. Coef.	t-value	P-value	Collinearity Statistics	
			B	S.E.	Beta			VIF	T

Constant			−128.11	31.67	-	−4.05	<0.001	-	-
NBME_Comb			6.14	0.55	0.60	11.27	<0.001	0.67	1.49
Com_Year_Bl_Avg			2.66	0.67	0.21	3.97	<0.001	0.58	1.73

**Table 5:** Stepwise linear regression model

	Unstandardized coefficients	Std. Error	Standardized coefficients		*t*-value	*P*-value
**Regression Step 1**						

(Constant)	−55.780	27.09	-		−2.05	0.040
Medicine	1.197	0.464	0.134		2.580	0.010
Surgery	1.257	0.396	0.158		3.173	0.002
Psychiatry	1.446	0.434	0.160		3.332	< 0.001
Obst_Gyn	2.228	0.416	0.263		5.356	< 0.001
Paediatrics	1.705	0.478	0.199		3.566	<0.001
Δ*R*^2^	0.592					

**Regression Step 2**						

**(**Constant)	−150.276	35.38	-		−4.25	< 0.001
Medicine	0.790	0.464	0.088		1.702	0.090
Surgery	0.877	0.399	0.110		2.199	0.028
Psychiatry	1.219	0.428	0.135		2.848	0.005
Obst_Gyn	1.853	0.417	0.219		4.446	< 0.001
Paediatrics	1.269	0.479	0.148		2.646	0.009
Year_1_Block_Avg	2.225	0.712	0.163		3.127	0.002
Year_2_Block_Avg	0.832	0.490	0.083		1.697	0.091
Δ*R*^2^	0.019					

**Regression Step 3**						

**(**Constant)	−159.127	44.13	-		−3.61	< 0.001
Medicine	0.694	0.469	0.078		1.482	0.139
Surgery	0.833	0.400	0.105		2.801	0.038
Psychiatry	1.244	0.429	0.137		2.903	0.004
Obst_Gyn	1.877	0.418	0.222		4.489	< 0 .001
Paediatrics	1.301	0.480	0.152		2.711	0.007
Year_1_Block_Avg	2.130	0.734	0.156		2.902	0.004
Year_2_Block_Avg	0.837	0.491	0.084		1.703	0.089
MCAT	3.983	2.70	0.055		1.473	0.141
GPA	−5.325	11.47	0.018		−0.46	0.643
Δ*R*^2^	0.002					
Total Model *R*^2^	0.613					

**ANOVA Table**^a^						
**Step**		**Sum of squares**	**d.f.**	**Mean square**	***F*-Value**	***P*-Value**

1	Regression	1096245.921	5	219249.184	104.423	< 0.001^b^
	Residual	757962.771	361	2099.620		
	Total	1854208.692	366			
2	Regression	1132336.258	7	161762.323	80.447	< 0.001^c^
	Residual	721872.435	359	2010.787		
	Total	1854208.692	366			
3	Regression	1136702.559	9	126300.284	62.842	< 0.001^d^
	Residual	717506.133	357	2009.821		
	Total	1854208.692	366			

a Dependent Variable: MCCQE-1

b Predictors: NBME subjects - Pediatrics, Psychiatry, Obstetrics and Gynecology, Surgery, Medicine

c Predictors: NBME subjects, Year 1 and 2 Block Average

d Predictors: NBME subjects, Year 1 and 2 Block Average, MCAT and GPA

**Table 6:** Evaluation of prediction model

	Actual MCCQE-1 exam performance			
Model prediction of MCCQE - 1		Pass	Fail	Total
	Pass	True Positive (a)	False Positive (b)	(a + b)
	Fail	False Negative (c)	True Negative (d)	(c +d)
	Total	(a +c)	(b + d)	(a + b + c + d)
